# Multi-device trust transfer: Can trust be transferred among multiple devices?

**DOI:** 10.3389/fpsyg.2022.920844

**Published:** 2022-08-03

**Authors:** Kohei Okuoka, Kouichi Enami, Mitsuhiko Kimoto, Michita Imai

**Affiliations:** ^1^Graduate School of Science and Technology, Keio University, Yokohama, Japan; ^2^Interaction Science Laboratories, ATR, Kyoto, Japan

**Keywords:** trust, human-agent interaction (HAI), trust transfer, human-AI cooperation, trusted AI, virtual agent, multi-device

## Abstract

Recent advances in automation technology have increased the opportunity for collaboration between humans and multiple autonomous systems such as robots and self-driving cars. In research on autonomous system collaboration, the trust users have in autonomous systems is an important topic. Previous research suggests that the trust built by observing a task can be transferred to other tasks. However, such research did not focus on trust in multiple different devices but in one device or several of the same devices. Thus, we do not know how trust changes in an environment involving the operation of multiple different devices such as a construction site. We investigated whether trust can be transferred among multiple different devices, and investigated the effect of two factors: the similarity among multiple devices and the agency attributed to each device, on trust transfer among multiple devices. We found that the trust a user has in a device can be transferred to other devices and that attributing different agencies to each device can clarify the distinction among devices, preventing trust from transferring.

## 1. Introduction

With the evolution of automation technology, autonomous systems, such as robots and self-driving cars, are becoming a familiar part of people's lives, and the opportunities for people and systems to work together are increasing. There are a variety of ways in which people and autonomous systems can collaborate, from having a person and an arm robot physically assemble parts together to having a person monitor a system while leaving the work to the system. Trust plays an important role in the collaboration between humans and autonomous systems because a system that cannot be trusted may lead to humans avoiding the system due to excessive risk perception of the work and miscommunication of intentions (Oleson et al., 2011).

Therefore, methods for estimating the trust people have in autonomous systems (John and Neville, 1992; Chen et al., 2020; Kohn et al., 2021) and methods for improving trust based on the estimated trust have been studied by investigating how people change their trust in autonomous systems (Floyd et al., 2015). Floyd et al. (2015) developed an algorithm to infer trust in oneself and generate more “trustworthy” behavior in robots working with humans. Soh et al. (2020) showed that when the same robot performs different tasks, the trust acquired in the previous task is transferred to the later task, a phenomenon called “multi-task trust transfer.” It was also shown that the degree of trust transfer is greater when both of the tasks are more similar and when the latter task is easier than the previous task.

Soh et al. have focused on the transition of trust in the same type of device. However, there are situations where multiple devices with different functions and shapes are operated (Tan et al., 2020, 2021). And, there are a lot of examples from the industrial situation such as the combination of trucks and excavators on a construction site (Stentz et al., 1999) to the daily situation such as the combination of smartphones and smartwatches (Chen et al., 2014). Also, even though multiple devices don't exist simultaneously, we could face the transition of trust among multiple devices such as when we update a device to a new one. Therefore, it is necessary to understand not only the transition of trust in the same type of device but also how trust transitions among multiple devices with different functions and shapes. We call the transition of trust among multiple devices “multi-device trust transfer (MDTT),” and investigated whether MDTT can occur and what characteristics it has through an experiment. This paper makes the following contributions:

- We conducted a human-subjects study and found that the trust a user has in a device can be transferred to other devices.
- We also found that creating different agents for each device can enhance the distinction among the devices, preventing trust from transferring.

## 2. Related work

### 2.1. Trust in human-robot interaction

The study of the trust humans have in robots is an important topic in human-robot interaction. However, trust is a multi-dimensional concept that has varying definitions even within the same field (Xie et al., 2019). For example, Gambetta (2000) defined trust as the subjective probability with which an agent assesses whether another agent will perform a particular action. Jones and Marsh (1997) broadly defined trust as being in three categories: basic trust, general trust, and contextual trust. Basic trust is the trust subject X has regardless of the object and is determined by X's experience. General trust is the trust X has in the other party Y regardless of the context. In a collaborative environment with an autonomous system, context is the task, and general trust is the trust X has in Y regardless of the task. Contextual trust is the trust X has in Y for a specific context, and in a collaborative environment, it is the trust X has in Y in task α. We focused on general trust and contextual trust to investigate the trust users have in an autonomous system.

The success or failure of a task affects trust (Chen et al., 2020). When a task succeeds, trust generally increases, and when it fails, trust decreases. Therefore, by repeating the same task, the user's trust in the system will converge to a value corresponding to the system's ability to perform the task. This process of adjusting the user's trust to a value suitable for the performance of the system is called “trust calibration” (Lee and See, 2004). When the user's trust is not properly calibrated and is lower than the actual performance of the robot, it is called “distrust,” and when it is higher than the actual performance, it is called “overtrust” (Lee and See, 2004). The uncalibrated state has various disadvantages (Oleson et al., 2011; Ullrich et al., 2021). For example, Freedy et al. (2007) showed that humans more frequently intervene with robots in the distrust state, resulting in longer work times. Therefore, several methods of facilitating calibration have been proposed (McGuirl and Sarter, 2006; Verberne et al., 2012; Okamura and Yamada, 2020; Zhang et al., 2020; Lebiere et al., 2021). For example, Wang et al. (2016) improved trust and performance by increasing the transparency of a robot using automatically generated descriptions of the robot.

### 2.2. Trust transfer

The observation of the success or failure of a task transfers not only to the observed task but also to different tasks. Soh et al. (2020) called this phenomenon “trust transfer” and investigated the effect of the differences in the relationships between tasks on it. In their experiment, they asked participants to observe a task in which an autonomous robot grasps an object and investigated how the user's trust in the same robot performing a different task changes before and after the observation.

Two factors, similarity between tasks and difference in difficulty, were varied in their experiment. For the similarity-between-tasks factor, they prepared two conditions of similar and dissimilar condition and used two types of tasks, a grasping task to grasp an object and a navigation task. In the similar condition, they evaluated the user's trust in the robot for the grasping task after observing the same task. Whereas, as the dissimilar condition, they evaluated it in the navigation task after observing the grasping task. For the difference-in-difficulty factor, they prepared two levels for each type of task, easy and difficult, by varying the ease of grasping the object in the grasping task and the presence or absence of an accompanying person in the navigation task.

The results of their experiment indicated that the degree of trust transfer was greater for the same type of task than for different tasks. It was also shown that observing the success of a task with high difficulty was transferred to increase the trust in a task with low difficulty. Soh et al. (2020) investigated trust transfer when the same device performed multiple tasks but did not investigate trust transfer among multiple devices. We investigated multi-device trust transfer (MDTT), which is trust transfer among multiple devices.

### 2.3. Agency transfer among multiple devices

In human-agent interaction, it has been shown that agency, the perception of intentionality in an anthropomorphic artifact, can transfer among multiple devices (Ogawa and Ono, 2008; Syrdal et al., 2009; Kim et al., 2013). In a system design in which a virtual agent, such as a computer graphics (CG) character, migrates to multiple devices, the agency of the virtual agent is also migrated to those devices. This agent is called a migrate agent.

Imai et al. (1999) have shown that using the ITACO system, in which the same on-screen agent between a display attached to the robot and a laptop, increased the impression of the robot among participants who interacted with the agent on the laptop. Reig et al. (2020) proposed a design in which a personal AI assistant on a user's smartphone transfers to service robots in public places. They compared their proposed design with that in which the robot does not adapt to the user and in which the robot adapts to the user by storing information about the user and found that users prefer the design where their personal AI assistant migrates to the robot.

Even though studies have suggested that agency can enhance trust in a system (Waytz et al., 2014; Large et al., 2019), previous research on migrate agents did not sufficiently investigate the effect of migration in terms of trust. Therefore, it is possible that the trust a user has in a migrate agent may also be transferred to the migrated devices along with the transfer of agency. Therefore, we also investigated MDTT when using a migrate agent and the relationship between agency and MDTT.

## 3. Experiment

We now describe our experiment designed to investigate the characteristics of MDTT. In particular, we formulated the following hypotheses and verified them through the experiment.

**Hypothesis 1 (H1):** When there are multiple devices and each device performs a task, the observation of one device's successful completion of the task will affect the trust in the other devices.

Research has been conducted on trust when several of the same devices are used, and it has been found that the behavior of one device affects the entire group (Gao et al., 2013; Fooladi Mahani et al., 2020). This suggests that trust may be transferred even among multiple devices with different functions and shapes.

**Hypothesis 2 (H2):** The degree of trust transfer is greater between similar devices than between devices with different characteristics.

It is thought that the more similar a device is to the observed device, the more likely trust will be transferred. For example, if we observe a self-driving car, we are more likely to apply that experience to another type of self-driving car, such as a bus, than to an autonomous drone.

**Hypothesis 3 (H3):** Using a migrate agent will increase the degree of trust transfer compared with not using it.

When agency transitions between devices using a migrate agent, it is thought that the user treats the source device and destination device as the same entity. As a result, trust is expected to be transferred along with the transition of the agency.

### 3.1. Overview

**Figure 1:**
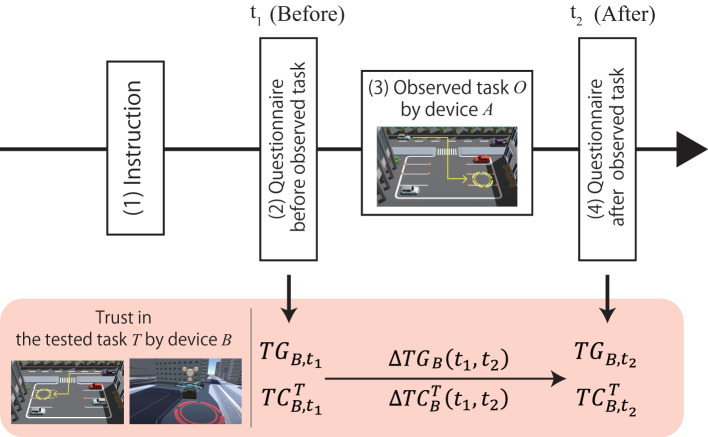
Experimental procedure proceeded from left to right, and *t*_*n*_ at top indicates when participants answered questionnaire. In lower part, correspondence between time and trust value is shown.

In this experiment, we used 3D CG to create videos of several different devices performing a task and investigated the characteristics of MDTT by measuring the trust in another device before and after the participants observed the video of one device performing the task. Each participant watched two videos depicting different devices performing a task. The task to be watched first is called the observed task, and the task to be watched after the observed task is called the tested task. Figure 1 shows the flow of this experiment, and the following sections provide details of the experimental settings. We measured trust transfer by evaluating the change in the trust in the device performing the tested task before and after the participants watch the observed task.

### 3.2. Conditions

We prepared the following three factors.

- **Time**: Time of evaluating trust

This is a factor to investigate the existence of MDTT (H1). Each condition in the time factor indicates the time when to evaluate the trust participants have in a device performing the tested task. There are two conditions: the “Before” condition, under which participants have not seen the observed task yet, and “After” condition under which participants have seen the observed task.

- **Device**: Similarity of devices

This is a factor to investigate the effect of device similarity on MDTT (H2). We prepared a “Similar-device” condition, under which the device performing the tested task has similar functionality to the device performing the observed task, and “Dissimilar-device” condition under which the device differs in functionality.

- **Agent**: Type of agents that migrate to devices

This is a factor to investigate the effect of agency transition on MDTT (H3). We prepared three conditions: the “No-agent” condition, under which no agent is created, “With-migrate-agent” condition, under which a migrating agent transfers among devices and performs two tasks, and the “With-agent” condition under which there is an agent for each device without transition.

To take into account the effect of viewing experience on trust, we designed the device and agent factors as between-subjects factors.

### 3.3. Experimental design

**Figure 2:**
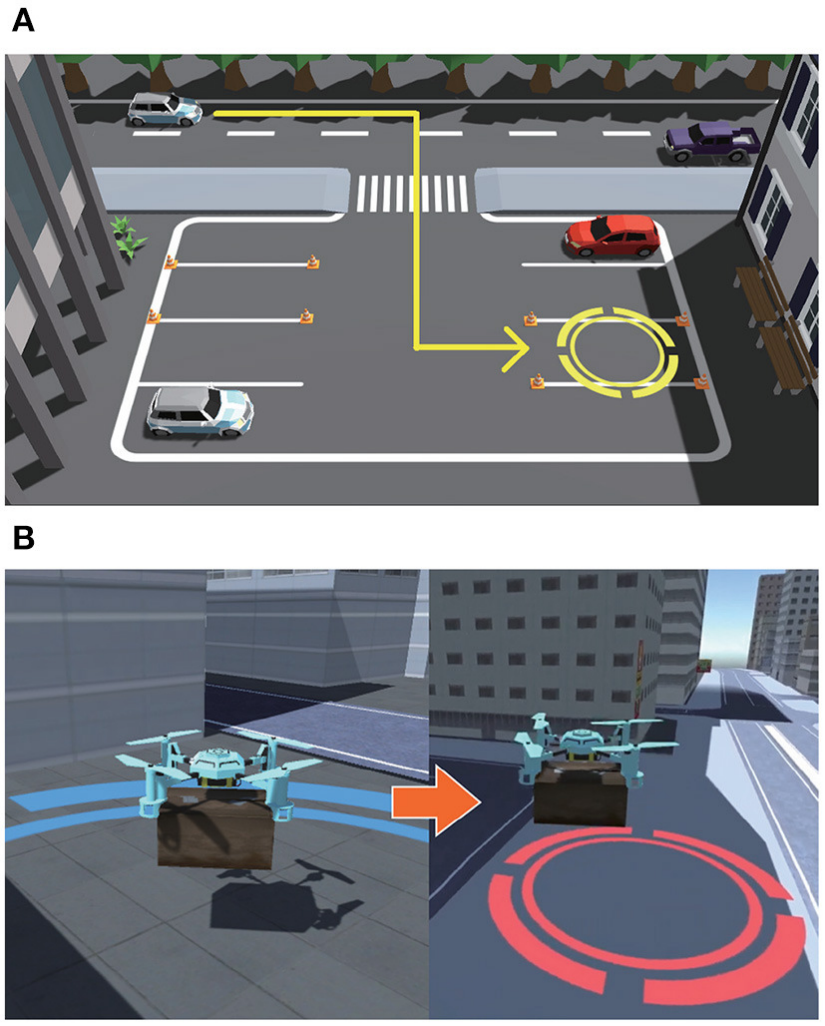
Scenes of two tasks used in this experiment. **(A)** Driving task. **(B)** Drone task.

The two tasks used in this experiment were a driving task and a drone task. The driving task involves a self-driving car parking in a parking lot, as shown in Figure 2A. The self-driving car at the top of the screen will attempt to park in the parking space indicated with the yellow circle. Cones are placed at the four corners of the parking space, and if the car hits one of these cones, the task will fail. The drone task, as shown in Figure 2B, involves a drone carrying luggage from the start point inside the blue circle through the city to the goal inside the red circles. If the drone collides with a building or another elevated structure during transportation, the task is considered a failure. We created videos of successful and failed scenes for each task.

The driving task was used as the observed task under all conditions and as the tested task under the Similar-device condition. The drone task was used under the Dissimilar-device condition. However, under the Similar-device condition, the observed and tested tasks used different types of vehicles and stop positions.

**Figure 3:**
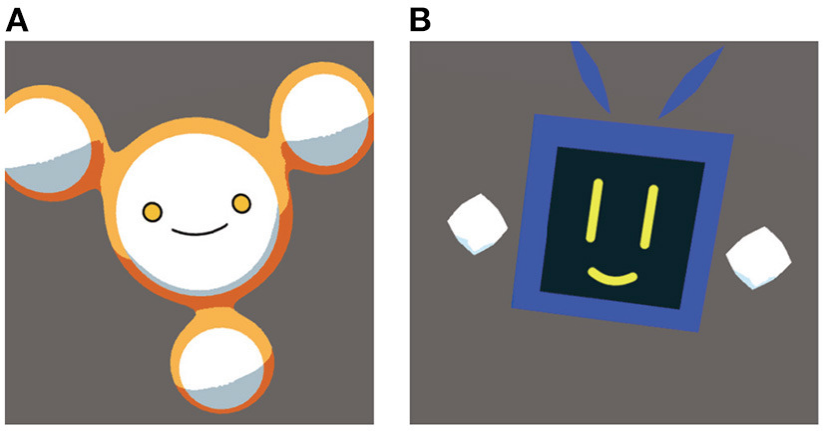
Design of agents. **(A)** Agent type “Yellow.” **(B)** Agent type “Blue.” These agents were used under with-agent and with-migrate-agent conditions.

Under the With-agent and With-migrate-agent conditions, we prepared two types of agents as shown in Figure 3. The name of each agent was “Blue” and “Yellow” based on their color, and the name was given under the condition where each agent was used. Different voices were used to clarify the differences between the agents. The agent was placed floating on the top of the device to make it clear that it is the subject that executes the task. Under the With-migrate-agent condition, we created a video to clarify that the agent is being transferred between devices.

### 3.4. Evaluation

To measure trust in a device, we created a questionnaire for trust evaluation referring to the questionnaire created by Washburn et al. (2020) and prepared two questions: “Can the robot be trusted?” and “Do you think this task will be successful?” Both questions were answered on a 7-point Likert scale. The former corresponds to the evaluation of general trust, and the latter corresponds to that of contextual trust.

We focused on the effect of the device and agent factors on not the existence but the degree of MDTT. Therefore, we introduced a scale called “trust change,” which was used in a previous study (Soh et al., 2020), to evaluate the degree of MDTT. Trust change is the degree of change in the trust value before and after the observation of a certain task. Let the value of general trust for device A at time *t* be *TG*_*A, t*_, and the value of contextual trust for device A in task L at time *t* be ${TC}_{A,t}^{L}$. Let *t*_*before*_ and *t*_*after*_ be the time before and after the observation of different devices performing the task, respectively, and the trust change for each trust Δ*TG*_*A*_ and ${\Delta TC}_{A}^{L}$ can be expressed as follows:


(1) $$\begin{array}{l} {\Delta TG}_{A}\left(t_{before},t_{after}\right)={TG}_{A,t_{after}}-{TG}_{A,t_{before}} \end{array}$$

(2) $$\begin{array}{l} {\Delta TC}_{A}^{L}\left(t_{before},t_{after}\right)={TC}_{A,t_{after}}^{L}-{TC}_{A,t_{before}}^{L} \end{array}$$

In short, to test H1, we used general trust and contextual trust and compared the difference between before and after seeing the observed task. To test H2 and H3, we converted both values into trust change and compared the difference due to the device and agent factors.

### 3.5. Procedure

Figure 1 shows the flow of the experimental procedure and corresponding evaluation values.

#### 3.5.1. Instructions

Participants first provided their age and sex then were explained the task they were going to watch during the experiment. Under the Similar-device condition, the driving task was explained, and under the Dissimilar-device condition, both driving and drone tasks were explained. When explaining the tasks, it was mentioned that the task can be successful or fail, and both successful and failed scenes were shown as examples.

After the explanation of the task, under the With-agent and With-migrate-agent conditions (not No-agent condition), the participants watched a video to instruct them that the agents will operate the devices. This video introduced the agents by their names and the task they were in charge of. Under the With-agent condition, two agents (Yellow and Blue) were introduced, and under the With-migrate-agent condition, only one of the agents was introduced. To understand how the trust questionnaire was asked to participants, we attached the detail of raw instruction as Supplementary material.

#### 3.5.2. Questions before observed task

Before watching the observed task, the participants were asked to answer the three questions related to trust and the execution time described in the previous section for both the observed and tested tasks. The time of the “Before” condition corresponds to this phase.

#### 3.5.3. Watching observed task

Participants watched the driving task being performed as an observed task. However, under the With-migrate-agent condition, the transition of the agent to the device used in the tested task was shown at the end of the video.

#### 3.5.4. Questions after observed task

After the participants finished watching the observed task, they were asked the same questions as in Section 3.5.2 for the observed and tested tasks. The time of the “After” condition corresponds to this phase. Then, according to the instruction that the participants would evaluate their trust in several tasks, participants watched the tested task and answered the questions as well as the observed task. Finally, participants were asked to answer the questions about the objects depicted in the video to avoid invalid responses and comment on the experiment as a whole by writing freely.

### 3.6. Participants

We recruited 600 participants using a crowdsourcing service. To investigate the effect of MDTT on various people, we prepared two criteria for recruiting participants. One of the criteria is the participants who are over 18 years old, the other is the participants who can read the Japanese instruction. There were 277 male participants (46.2%), 320 female participants (53.3%), and 3 participants who answered “other” (0.5%), and the average age was 39.7 years old. 100 participants were equally assigned to each of the six conditions, which were a combination of device and agent factors. Each participant was paid 135 yen equally as a reward after the experiment through that service. In order to eliminate the influence of the agent's appearance, the With-migrate-agent condition was designed so that the number of participants in the experiment for each agent, Yellow and Blue, was the same. Under the With-agent condition, we made the number of participants watching the video that each agent performs the observed task equally.

### 3.7. Results

#### 3.7.1. Time factor

**Figure 4:**
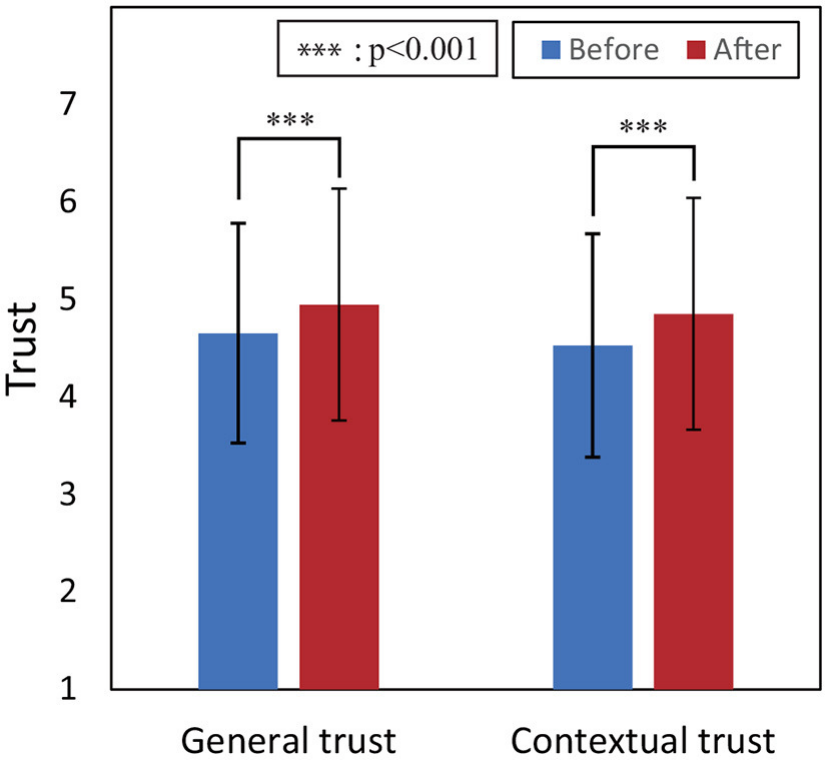
Results of comparison between mean of each trust value before and after watching observed task. Error bars represent standard deviations.

Figure 4 shows the values of general trust and contextual trust for the tested task before and after watching the observed task. To statistically test the differences in these values, a three-way analysis of variance (ANOVA) was conducted, taking into account the effect of the device and agent factors, and adding the both factors in addition to the time factor of before and after watching.

Regarding contextual trust, there was a significant difference in the time factor were shown at a 5% level of significance [*F*_(1, 594)_ = 70.45, *p* < 0.001, partial η^2^ = 0.11]. And there was no three-way and two-way interaction, so it was shown that contextual trust increased after the viewing of the observed task in all conditions of device and agent factor.

Regarding general trust, there was a significant difference in the time factor [*F*_(1, 594)_ = 80.47, *p* < 0.001, partial η^2^ = 0.12]. However, there was a two-way interaction between the time and agent factors [*F*_(2, 594)_ = 3.57, *p* = 0.029 < 0.05, partial η^2^ = 0.012]. There was no need to test for the simple main effect of the agent factor under each time condition; thus, we tested for the simple main effect of the time factor under each agent condition followed by Bonferroni correction. We found that the simple main effect of the time factor under all agent conditions was significant (No-agent: *p* < 0.001; With-agent: *p* = 0.001; With-migrate-agent: *p* < 0.001). This suggests that general trust significantly increased after the viewing of the observed task. These results indicate that both contextual trust and general trust significantly increased before and after the watching of the observed task regardless of device and agent factors, supporting H1.

#### 3.7.2. Device and agent factors

**Figure 5:**
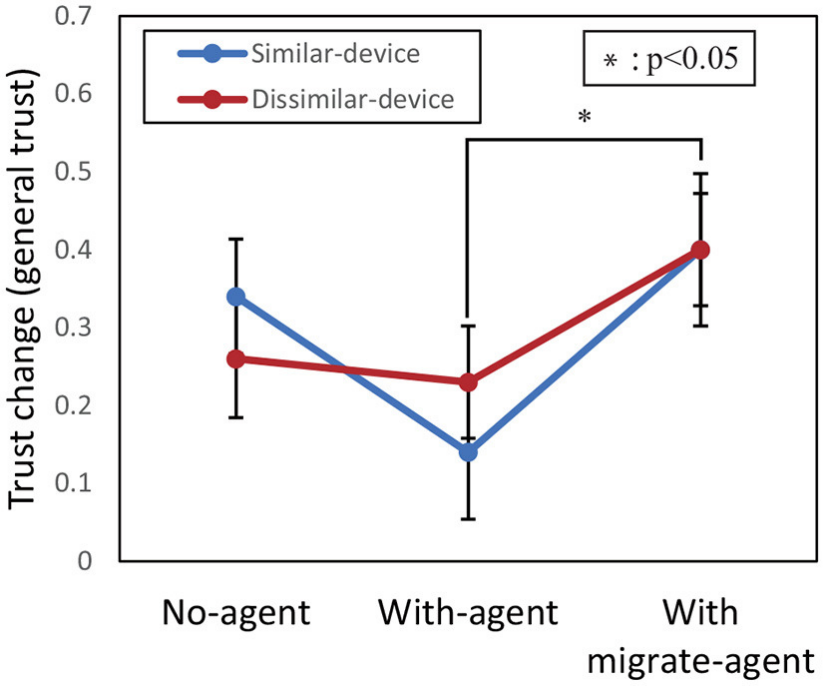
Results of comparison between mean scores of trust change of general trust. Error bars represent standard errors.

**Figure 6:**
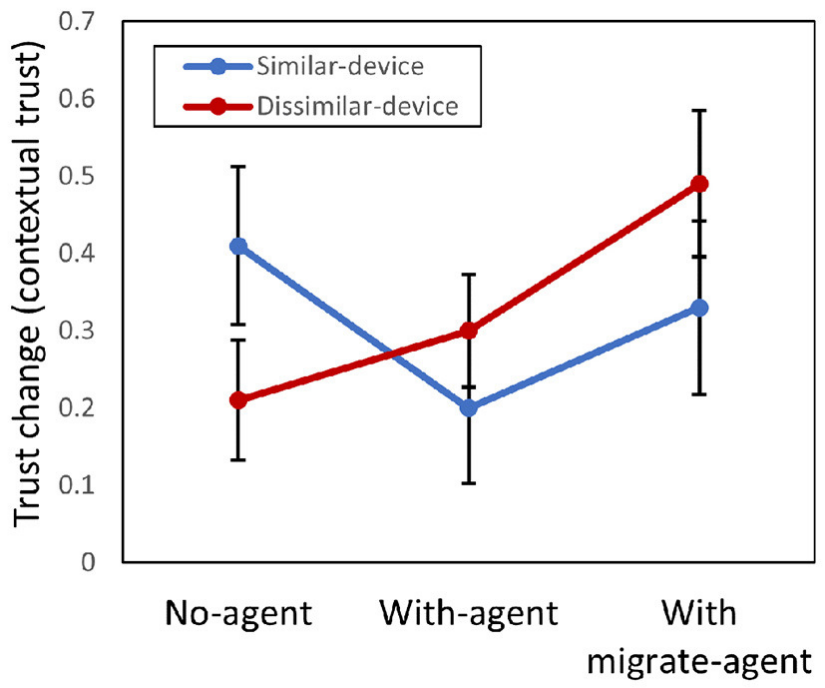
Results of comparison between mean scores of trust change of contextual trust. Error bars represent standard errors.

To evaluate the effect of the device and agent factors on the degree of MDTT, a two-way ANOVA between the device and agent factors was conducted on the trust-change values of general trust (Figure 5) and contextual trust (Figure 6).

Regarding general trust, there was a significant difference in the agent factor [*F*_(2, 594)_ = 3.57, *p* = 0.029, partial η^2^ = 0.012]. However, there were no significant differences for the device factor [*F*_(1, 594)_ = 0.003, *p* = 0.96, partial η^2^<0.001] and a two-way interaction between both factors [*F*_(2, 594)_ = 0.58, *p* = 0.57, partial η^2^ = 0.002]. Therefore, multiple comparisons using the Bonferroni method for the agent factor showed a significant difference between the With-migrate-agent and With-agent conditions (*p*=0.023).

Regarding contextual trust, there was no significant difference in the agent factor [*F*_(2, 594)_ = 1.47, *p* = 0.231, partial η^2^ = 0.005], device factor [*F*_(1, 594)_ = 0.067, *p* = 0.79, partial η^2^<0.001], and a two-way interaction between both factors [*F*_(2, 594)_ = 2.09, *p* = 0.13, partial η^2^ = 0.007].

The results show that H2, “The degree of trust-transfer will be greater between similar devices than between devices with different properties,” was rejected because there was no difference between the device factor regarding trust change for both general trust and contextual trust.

For H3, “Using a migrate agent increases the degree of trust transfer compared with not using a migrate agent,” it was found that the With-migrate-agent condition increased the degree of trust transfer compared with the With-agent condition regarding general trust. However, since there was no significant difference compared with the No-agent condition, H3 was partially supported.

## 4. Discussion

### 4.1. Implications

We investigated the characteristics of MDTT using videos including 3D CG characters. Hypothesis 1 was supported by the result that both general trust and contextual trust were significantly higher after watching than before watching the observed task. This was also supported by the result that the after-watching ratings were significantly higher regardless of device and agent factors, indicating that MDTT occurred under all conditions. When multiple autonomous devices are used at the same time, the trust that the user has in one device transitions to another device, so it is necessary to take the other devices into account when estimating the trust in a device. This is especially important to accurately estimate trust when a user has little experience in observing a device's ability to perform a task, such as when using a new system.

Hypothesis 2 was rejected because no difference was found in the device factor between general trust and context trust. This suggests that device similarity has no effect on the degree of MDTT. However, in a previous study of trust transfer (Soh et al., 2020), the similarity of tasks enhanced the degree of the trust transfer. As a hypothesis for the difference, the trust transfer might be weakened by transfer among multiple different devices. As the result, the effect of similarity was also weakened and lost. From the free descriptions for the Dissimilar-device condition, the comment “I thought the drone would also work well because the parking was smooth” was obtained, suggesting that MDTT may occur even when devices are dissimilar in properties. Although two types of tasks were used, driving and drone, there were comments that both were broadly regarded as automatic-driving tasks, which may be due to the fact that the properties of the devices were similar to some extent even under the Dissimilar-device condition.

Although there was no significant difference between the No-agent and With-migrate-agent conditions regarding H3, the degree of trust transfer was significantly smaller under the With-agent condition in terms of general trust. This means that the transition of agency does not increase the degree of MDTT, but attributing different agencies to each device weakens MDTT. In Soh et al.'s (2020) research, the value of general trust was correlated with that of contextual trust. Thus, this unbalanced result is unintuitive. As a hypothesis for the difference, among the multi-dimensions of trust, migrate agents might specifically affect the dimension related to personality. The general trust is evaluated for the system regardless of a task, thus the evaluation of it might be near to that of personality. And, it is shown that migrate agents could increase the impression (Imai et al., 1999). Therefore, migrate agents increased the general trust. On the other hand, when using a device with each agent, it emphasized the distinction between agents and prevented the transfer of general trust clearly. It is also possible to avoid overtrust and negative trust transfer by intentionally changing the agency to be attributed to each device. Also, the other reason there was no difference between the No-agent and With-migrate-agent conditions is that even under the No-agent condition, identification is possible between devices. We did not explicitly indicate in the instructions that the device performing the observed task and that performing the tested task were different.

### 4.2. Limitations

We created videos using 3D CG and had people watch them. However, people may have different impressions of a physical device and 3D CG, which may affect the transition of trust. Therefore, it is necessary to conduct experiments to investigate the effects of using actual devices. We investigated MDTT when the observed task was successful, but in reality, a system may fail. In previous studies (Lee and See, 2004), the failure of a task decreased trust. Therefore, it is necessary to investigate negative MDTT since the decrease in trust transfer due to failure of the observed task may be transferred to another device. It is also necessary to consider more types of devices, such as humanoid robots, and investigate the effects of such differences in more detail.

In this experiment, we designed the agents and devices with reference to the previous work (Reig et al., 2020) so that the participants can distinguish between the device and agent of the observed task and that of the tested task. For the identification, Reig et al. changed the name, voice, and appearance of a virtual agent. Therefore, in our experiment, we designed the two agents to have different names, voices, and appearances each other. Also, for the device, we designed different appearances and names for each device. Under the Similar-device condition, one is a light blue compact car, and the other is a blue truck. Under the Different-device condition, one is a light blue compact car, and the other is a light blue drone. According to the above setting, we designed the appearance and explained those devices in the instructions. However, since we did not execute the manipulation check for the participant's device identification, there is the possibility that the participants did not distinguish between the device of the observed task and that of the tested task, and the participants transferred their trust in the device because they recognized it as the same one.

## 5. Conclusion

We formulated three hypotheses regarding multi-device trust transfer (MDTT), the transition of trust among multiple different devices and conducted an experiment on MDTT. We found that by observing one device successfully completing a task, trust is transferred to other different devices. However, there was no effect on the degree of MDTT due to differences in the similarity of devices. As a result of investigating the effect of the transition of agency by a migrate agent on the degree of MDTT, it was found that there was no difference in this degree when comparing the case in which a migrate agent was used and in which no agent was used. However, compared with drawing a different agent for each device, the degree of MDTT became larger when using a migrate agent.

## Data availability statement

The original contributions presented in the study are included in the article/Supplementary material; further inquiries can be directed to the corresponding author.

## Ethics statement

The studies involving human participants were reviewed and approved by Keio University Faculty of Science and Technology, Graduate School of Science and Technology Bioethics Committee. All participants in this study read our informed consent presented in web form and approved the participation.

## Author contributions

KE conducted the experiment. KO performed the statistical analysis and drafted the manuscript. All authors contributed to the conception and design of the study, manuscript revision, read, and approved the submitted version.

## Funding

This work was supported in part by JST, CREST Grant Number JPMJCR19A1, Japan and JSPS KAKENHI Grant Number JP20K19897.

## Conflict of interest

The authors declare that the research was conducted in the absence of any commercial or financial relationships that could be construed as a potential conflict of interest.

## Publisher's note

All claims expressed in this article are solely those of the authors and do not necessarily represent those of their affiliated organizations, or those of the publisher, the editors and the reviewers. Any product that may be evaluated in this article, or claim that may be made by its manufacturer, is not guaranteed or endorsed by the publisher.
