# Supplementary material for: Multi-device trust transfer: Can trust be transferred among multiple devices?
Source: Front Psychol. 2022 Aug 3;13:920844. doi: 10.3389/fpsyg.2022.920844 (PMC9382300; doi:10.3389/fpsyg.2022.920844)
Supplement: Supplementary file 1 [file Data_Sheet_1.PDF]

frontiers

Supplementary Material

1 INSTRUCTION OF THE EXPERIMENT

In the experiment, we presented the instructions for the pariticipants before answering the questionnaire. In the following sections, we will show the raw instruction under each condition. Also, Table S1 shows the corresponding between the setting of conditions and the instructions flow. But, please note that this is the instruction translated from the original Japanese into English.

Table S1. The correspondence table between the setting of conditions and the instructions flow

Device	Agent	Instruction flow
Similar-device	No-agent	Section1.1
	With-agent	Section1.1 → Section1.3
	With-migrate-agent	Section1.1 → Section1.4
Dissimilar-device	No-agent	Section1.2
	With-agent	Section1.1 → Section1.3
	With-migrate-agent	Section1.1 → Section1.4

1.1 For the Similar-device condition

Thank you for your cooperation in our experiment. We will explain about the content of this experiment. In this experiment, we investigate the user's trust in self-driving car.

You will watch a video that various self-driving cars park in a parking lot. The task will be successful if the cars can reach the parking position marked in yellow without climbing over the curb or hitting the cones. However, the system is not perfect. Parking can be a success or a failure. You will be asked to predict for each self-driving car whether or not it will succeed in parking. In this experiment, you will see two self-driving cars park. The first is a light blue compact car, and the second is a blue truck.

1.2 For the Dissimilar-device condition

Thank you for your cooperation in our experiment. We will explain about the content of this experiment. In this experiment, we investigate the user's trust in the system. You will be asked to evaluate two different tasks.

In the first task, the self-driving car parks in a parking lot. The task is successful if the car can reach the parking position marked in yellow without running up a curb or hitting a set of cones. However, the system is not perfect. Parking can be a success or a failure. You will be asked to predict whether the car will be successful or not. In the actual experiment, you will see a light blue compact car park.

In the second task, the drone will transport the cargo. The task will be successful if the drone dives under the elevated road and elevated railroad tracks and reaches the red finish line from the blue start point. This system is not perfect either, so sometimes it succeeds and sometimes it fails. As well as the first task, the participants will be asked to predict whether the drone will succeed in transporting the cargo or not. In the actual experiment, you will see the light blue drone transport the cargo.

1.3 For the With-agent condition

The system will appear as a computer-graphcis character. After this instruction, please watch their greet.

(Agent that is in charge of the observed task)
Nice to meet you. I'm in charge of the first task, my name is Yellow / Blue, a task assist robot. I will do my best, so thank you.

(Agent that is in charge of the tested task)
Nice to meet you. I'm in charge of the second task, my name is Yellow / Blue, a task assist robot. I will do my best, so thank you.

1.4 For the With-migrate-agent condition

The system will appear as a computer-graphcis character. After this instruction, please watch their greet.

(Agent)
Nice to meet you. I'm in charge of the tasks in this experiment, my name is Yellow / Blue, a task assist robot. I will do my best, so thank you.
